# Restored Collagen VI Microfilaments Network in the Extracellular Matrix of CRISPR-Edited Ullrich Congenital Muscular Dystrophy Fibroblasts

**DOI:** 10.3390/biom14111412

**Published:** 2024-11-06

**Authors:** Daniela Benati, Eleonora Cattin, Federico Corradi, Tommaso Ferrari, Eleonora Pedrazzoli, Clarissa Patrizi, Matteo Marchionni, Roberto Bertorelli, Veronica De Sanctis, Luciano Merlini, Alessandra Ferlini, Patrizia Sabatelli, Francesca Gualandi, Alessandra Recchia

**Affiliations:** 1 Department of Life Sciences, University of Modena and Reggio Emilia, 41125 Modena, Italy; daniela.benati@unimore.it (D.B.); eleonora.cattin@unimore.it (E.C.); federico.corradi@unimore.it (F.C.); tommaso.ferrari@unimore.it (T.F.); eleonora.pedrazzoli@fht.org (E.P.); clarissa.patrizi2@gmail.com (C.P.); matteo.marchionni@studenti.unimore.it (M.M.); 2 Next Generation Sequencing Core Facility Department of Cellular, Computational and Integrative Biomedicine—CIBIO LaBSSAH, University of Trento, 38122 Trento, Italy; roberto.bertorelli@unitn.it (R.B.); veronica.desanctis@unitn.it (V.D.S.); 3 Department of Biomedical and Neuromotor Science, DIBINEM, University of Bologna, 40136 Bologna, Italy; mrllcn@unife.it; 4 Section of Medical Genetics, Department of Medical Sciences, University of Ferrara, 44121 Ferrara, Italy; fla@unife.it; 5 Unit of Medical Genetics, Department of Mother and Child, University Hospital S. Anna Ferrara, 44121 Ferrara, Italy; gdf@unife.it; 6 CNR-Institute of Molecular Genetics, 40136 Bologna, Italy; sabatelli@area.bo.cnr.it; 7 IRCCS Istituto Ortopedico Rizzoli, 40136 Bologna, Italy

**Keywords:** CRISPR/Cas9, allele-specific gene editing, collagen VI, collagen VI-related disorders, patient-derived fibroblasts

## Abstract

Collagen VI is an essential component of the extracellular matrix (ECM) composed by α1, α2 and α3 chains and encoded by *COL6A1*, *COL6A2* and *COL6A3* genes. Dominant negative pathogenic variants in *COL6A* genes result in defects in collagen VI protein and are implicated in the pathogenesis of muscular diseases, including Ullrich congenital muscular dystrophy (UCMD). Here, we designed a CRISPR genome editing strategy to tackle a dominant heterozygous deletion c.824_838del in exon 9 of the *COL6A1* gene, causing a lack of secreted collagen VI in a patient’s dermal fibroblasts. The evaluation of efficiency and specificity of gene editing in treating patient’s fibroblasts revealed the 32% efficiency of editing the mutated allele but negligible editing of the wild-type allele. CRISPR-treated UCMD skin fibroblasts rescued the secretion of collagen VI in the ECM, which restored the ultrastructure of the collagen VI microfibril network. By using normal melanocytes as surrogates of muscle cells, we found that collagen VI secreted by the corrected patient’s skin fibroblasts recovered the anchorage to the cell surface, pointing to a functional improvement of the protein properties. These results support the application of the CRISPR editing approach to knock out *COL6A1* mutated alleles and rescue the UCMD phenotype in patient-derived fibroblasts.

## 1. Introduction

Collagen VI is a structural component of the extracellular matrix (ECM) composed by α1, α2 and α3 chains, which are encoded by three genes (*COL6A1*, *COL6A2* and *COL6A3*). The assembling process to form mature collagen VI protein begins with the association of the three α-chains to form a triple-helix (TH) monomer followed by the formation of antiparallel dimers that lastly align to form large tetramers. After post-translational modifications, the tetramers are secreted in the ECM to form the characteristic beaded microfilaments that play a remarkably broad range of key roles in different tissues [1]. These include cytoprotection by counteracting apoptosis and oxidative damage, regulation of autophagy and cell differentiation, contribution to stem cell self-renewal and tissue regeneration, and promotion of tumor growth and progression [2].

Mutations in collagen VI genes (*COL6A1*, *COL6A2* and *COL6A3*) in humans result in decreased, lack of or aberrant collagen VI protein leading to the deterioration of the extracellular matrix structure, increased apoptosis and oxidative stress, and decreased autophagy and impaired muscle regeneration [3]. These defects are common to a broad spectrum of rare muscular and neurological diseases, including Bethlem myopathy (BM, OMIM 158810) and Ullrich congenital muscular dystrophy (UCMD, OMIM 254090) [4,5,6]. Bethlem myopathy is the mildest condition characterized by axial and proximal muscle weakness with prominent distal contractures. UCMD is a severe disorder characterized by congenital muscles, weakness, proximal contractures and distal laxity. Moreover, patients affected by collagen VI-related myopathies show joint contractures and distal joint hypermobility due to alterations of the tendon matrix affecting collagen VI, I and XII [7].

UCMD fibroblasts isolated from the skin, and differentially from tendons, do not exhibit defects in the collagen VI-NG2 (neural/glial antigen 2) axis that affects cell polarization during migration in response to in vitro injuries [7] and do not show the mitochondria defects [8] observed in skeletal muscle fibers and myoblast cultures of Col6a1−/− mice and in muscle cell cultures from patients affected by ColVI myopathies [9]. However, UCMD skin fibroblasts display alterations of the collagen VI network in the ECM mimicking the sarcolemma-specific collagen VI deficiency status in UCMD muscles. Based on this defect, UCMD skin fibroblasts have been used to test different gene therapy approaches.

Small interference RNA to silence the *COL6A3* allele carrying an in-frame deletion in exon 16 [10] or to specifically silence the p.G284R or p.G293R point mutations in the *COL6A1* gene [11,12] have been demonstrated to considerably improve the quantity and quality of the collagen VI matrix in transfected skin fibroblasts from UCMD patients.

As alternative transient repressors, antisense oligonucleotides (AON) were used to correct UCMD patient fibroblasts hosting a de novo dominant mutation in intron 9 of the *COL6A2* gene (c.954+17_954+22del28) [13]. Similarly, gapmer AON silenced a mutated allele carrying a de novo 18-nt genomic deletion in exon 15 of the *COL6A3* gene, resulting in reduced intracellular retention of the collagen VI complex and increased secretion of a functional protein in the ECM [14]. More recently, AON efficiently mediated the skipping of a pseudoexon generated by the de novo recurrent deep intronic mutation c.930+198C > T in *COL6A1* gene [15]. This mutation in the *COL6A1* gene was also tackled by the Bonnemann group who employed phosphorodiamidate morpholino oligomers (PMOs) and 2′-O-methoxyethyl (MOE) phosphorothioate oligomer to promote exon skipping or a SpCas9 combined to two gRNAs to delete the intronic mutation. A marked reduction of the mutant chain and an unchanged level of total collagen VI was observed, suggesting that the normal splicing of both alleles was not affected [16]. A CRISPR approach based on mutation-specific gRNA was also proposed to permanently knock down the dominant-negative pathogenic variants, p.Gly293Arg [17] or p.Gly290Arg [18], of collagen α1 caused by the missense mutation c.877G> A or c.868G> A, respectively, in exon 10 of the *COL6A1* gene. These variants have been often associated with a reduced secretion of collagen VI in the ECM compared to healthy donor (HD) fibroblasts. To correct the G293R variant, Lopez-Marquez et al. set up an HDR-based approach consisting of SpCas9 combined to two gRNAs in the ribonucleoprotein complex (RNP). Although the HDR frequency was less than 0.5% in most of the allelic variants, a relevant knockdown of the mutant allele caused by either gRNA1 or gRNA2 was reported with almost undetectable editing on the WT allele. CRISPR-treated fibroblasts showed transcriptional silencing of the mutant allele and recovered the secretion of collagen VI positive fibers [17]. The other missense mutation in exon 10 of the *COL6A1* gene (G290R) was recently tackled by Bolduc et al. with a regular WT SpCas9 nuclease plasmid combined to a relaxed gRNA harboring intentional mismatches close to the variant to reduce the targeting of the WT allele. This strategy resulted in frameshifting the c.868G> A allele and an increase of collagen VI secretions in the ECM, thus supporting the haploinsufficiency of the *COL6A1* gene [18].

In this study, we tackled the p.Gly275_Lys280del variant caused by dominant heterozygous deletion in exon 9 (c.824_838del) of the *COL6A1* gene. This mutation, less frequent than the missense ones [19], dramatically impacts the formation of the TH monomer and results in a marked reduction of the expression of collagen VI in muscle biopsies [20]. As per the CRISPR approach, we exploited high-fidelity (HF) Alt-R SpCas9 complexed to a single gRNA specific to the mutant allele, thus reducing the possible off-target editing and the risk of rearrangements frequently associated with the introduction of two double-strand breaks [21].

Here, we showed that dermal-derived patient fibroblasts, which represent a common cell model for collagen VI secretion [22], do not secrete collagen VI in the ECM. The proposed CRISPR approach allowed highly specific and safe targeting of the pathogenic allele and silencing of the mutant transcript. Upon treatment with CRISPR RNP, the UCMD fibroblasts secreted mature collagen VI in the ECM, which is comparable to HD fibroblasts. A fine characterization of the ultrastructure of the collagen VI microfibril network showed the formation of long-branched polymers. Lastly, collagen VI secreted from a bulk population of CRISPR-corrected skin fibroblasts recovered the anchorage to the cell surface, as demonstrated by the restoration of the collagen VI-NG2 axis in normal melanocytes.

## 2. Materials and Methods

### 2.1. Plasmids

The CRISPR/Cas9 effector plasmids for gRNA1 and gRNA3 were generated by cloning two annealed oligonucleotides for each gRNA into *BbsI* sites of the pX330-U6-Chimeric_BB-CBh-hSpCas9 plasmid (Addgene plasmid # 42230). The CRISPR/Cas9 effector plasmid for gRNA2 was obtained by cloning annealed oligonucleotides in the pX330-U6-Chimeric_BB-CBh-hSpCas9 plasmid into *BbsI* sites, followed by subcloning of the U6-gRNA2 cassette into the MSP2440 plasmid (Addgene plasmid # 72250) carrying the expression cassette for VQRHF1-SpCas9 [23]. To generate template plasmids carrying the wild-type (WT) or c.824_838del pathogenic variant in exon 9 of COL6A1, genomic DNA from UCMD patient fibroblasts was extracted with a QIAamp DNA Micro Kit (Qiagen, Venlo, The Netherlands), and the COL6A1 target region was amplified with COL6A1_F and COL6A1_R primers (Table S1), using the GoTaq G2 Green Master Mix (Promega, Fitchburg, WI, USA) according to the manufacturer’s instructions. The PCR products from both alleles were separated in a 2% agarose electrophoresis gel, and isolated bands from COL6A1 WT and mutated alleles were separately cloned in a pCR 2.1-TOPO TA vector (Thermo Fisher Scientific, Waltham, MA, USA), according to the manufacturer’s instructions. The resulting TOPO TA vectors carrying either the WT or mutated fragments were Sanger sequenced.

### 2.2. Cell Culture

HEK293T cells were obtained from the American Type Culture Collection (ATCC #CRL-3216) and were cultured in Dulbecco’s modified Eagle’s medium (DMEM) supplemented with 10% fetal calf serum (FCS), 100 U/mL penicillin and 100 mg/mL streptomycin (Lonza Ltd., Basel, Switzerland). Genetic data for the UCMD patient carrying the heterozygous c.824_838del pathogenic variant in exon 9 of COL6A1 were published previously (pt.2 in [20]). Patient fibroblast collection for genomic study was approved by the UNIFE Ethical Committee with approval no. EM434-2020_AOUFe/161299_EM1 (20/05/2020). The patient provided written informed consent. Normal melanocytes and fibroblasts from healthy subjects were obtained after written informed consent and Ethics Committee approval at the Rizzoli Orthopedic Institute (project identification code: CE 0021392, 9 November 2016 and CE 0007151, 11 May 2021). Primary fibroblasts from the UCMD patient or healthy donor were established from skin biopsies and cultured at 37 °C and 5% CO2 in DMEM enriched with 20% fetal bovine serum (FBS), 1% Penicillin/Streptomycin and 1% Glutamine. The ColVI-enriched conditioned medium was obtained by treating confluent skin fibroblast cultures from a healthy donor and from the UCMD patient before and after editing with DMEM supplemented with 0.25 mM L-ascorbic acid. After 24 h, the medium was collected, centrifuged at a low speed to remove cell debris, and diluted 1:1 with M254 medium. Melanocytes from a healthy subject were plated onto coverslips and maintained in a M254 culture medium (GIBCO, Thermo Fisher Scientific, Waltham, MA, USA) supplemented with an HMGS supplement (GIBCO, Thermo Fisher Scientific, Waltham, MA, USA) until they reached 70% confluence and treated with ColVI-enriched media for 24 h before immunohistochemical analysis with anti-ColVI and anti NG2 antibodies (Merck, Kenilworth, NJ, USA).

### 2.3. Transfections of Cells

The transfection of 3.2 × 10^5^ HEK293T cells was performed using CaPO_4_ protocol with 1.5 μg of template plasmids and 3 μg of effector plasmids. The electroporation of primary fibroblasts with Alt-R ribonucleoproteins (RNP) was performed as described in Martufi et al. [24]. Briefly, Alt-R CRISPR-Cas9 crRNAs and tracrRNA (IDT Integrated DNA Technologies, Coralville, IA, USA) were resuspended in nuclease-free duplex buffer (IDT Integrated DNA Technologies, Coralville, IA, USA) at a concentration of 100 μM, and mixed in equal molar amounts at a concentration of 25 μM, as recommended by the manufacturer. To generate RNP, 72.5 pmol of annealed crRNA–tracrRNA were mixed with 60 pmol of Alt-R S.p. HiFi Cas9 Nuclease V3 (IDT Integrated DNA Technologies, Coralville, IA, USA) and incubated for 10 min at room temperature. Next, 60 pmol of Alt-R Cas9 Electroporation Enhancer (IDT Integrated DNA Technologies, Coralville, IA, USA) was added, as recommended by the manufacturer. Primary fibroblasts were resuspended in a 95.5 μL P3 solution using a P3 Primary Cell 4D-Nucleofector Kit L (Lonza Ltd., Basel, Switzerland), mixed with 4.5 μL of Alt-R RNP, and electroporated using program CM-138.

### 2.4. Analysis of CRISPR/Cas9 on- and Off-Target Editing

To analyze editing efficiency and specificity in transfected HEK293T cells, DNA was extracted using a QIAamp DNA mini kit (QIAGEN, Hilden, Germany) 48 h post-transfection, following the manufacturer’s instructions. WT or mutated COL6A1 regions on template plasmids were amplified by PCR using Platinum Superfi II DNA polymerase (Thermo Fisher Scientific, Waltham, MA, USA) and primers, as in Table S1, and analyzed with TIDE webtool [25] to evaluate the indels frequency. For NGS analysis of on-target editing in primary fibroblasts, genomic DNA was extracted 48 h post-electroporation, using a QIAamp DNA micro kit (QIAGEN, Hilden, Germany), according to the manufacturer’s protocol. The genomic regions flanking gRNA target sites were amplified by PCR using Platinum Superfi II DNA polymerase (Thermo Fisher Scientific, Waltham, MA, USA) and primers, as in Table S1. Illumina Nextera barcodes were added to amplicon libraries by a limited number (*n* = 8) of PCR cycles using “2nd amplification” primers, as in Table S1. Libraries were purified using a QIAGEN PCR purification kit (QIAGEN, Hilden, Germany). Equimolar amounts of libraries were mixed, diluted, and sequenced using the Illumina MiSeq system (Illumina Inc., San Diego, California, USA) (paired-end sequencing; 2 × 250-bp). To analyze CRISPR editing on WT or c.824_838del COL6A1 alleles from the heterozygous UCMD patient, NGS reads were segregated according to C > T SNP located 62 nt downstream at the end of exon 9 of the WT allele using a custom Python script (v.3.10.12) (WT sequence GGATCTCCGGCTTCTCCCTT; mutant sequence GGATCTCCGGCTTCTCCCTC). After segregation, reads for the WT or mutated alleles were separately analyzed by CRISPResso2 [26]. The off-target analysis for each gRNA was performed by using the COSMID [27] webtool. In the search options of COSMID, three mismatches were allowed without InDels, and one mismatch was allowed in the presence of one deletion or insertion. Off-target sites with scores lower than 10 were PCR amplified using Platinum Superfi II DNA polymerase (Thermo Fisher Scientific, Waltham, MA, USA) and primers, as in Table S1. Amplicon libraries were barcoded, as described above, purified with a QIAquick PCR Purification Kit (QIAGEN, Hilden, Germany), and sequenced using the Illumina MiSeq system (paired-end sequencing; 2 × 250-bp). Targeted deep sequencing data were analyzed using CRISPResso2 [26].

### 2.5. Transcript Analysis of COL6A Genes

Total RNA, purified from primary fibroblasts electroporated or not with Alt-R RNPs, was isolated with an RNeasy Micro kit (QIAGEN, Hilden, Germany) according to the manufacturer’s instructions. The synthesis of cDNA was performed as previously described [28]. Primers and allele-specific probes (Table S1) were designed to measure exon 9 WT and mutated *COL6A1* transcripts by droplet digital PCR (ddPCR) with the Bio-Rad platform (Hercules, CA, USA). The PCR was set up according to manufacturer protocol and was amplified with the following cycling conditions: 95 °C for 10 min, 39 cycles of 94 °C for 30 s and 55 °C for 1 min for annealing and extension and 10 min at 98 °C for reaction termination. The PCR plate was then placed into a Q × 200 droplet reader for data analysis. The wild-type and mutant alleles were distinguished by a two-dimensional view of the ddPCR analysis and analyzed by using Bio-Rad QX Manager software 1.2 Standard Edition. Five biological replicates were carried out for each experiment. For the quantification of total *COL6A1* transcripts, a probe specific to the human_COL6A1 (assay HS01095585_m1) and a probe specific to housekeeping were used. The ddPCR was performed as indicated above. The PCR plate was amplified with the following cycling conditions: 95 °C for 10 min, 39 cycles of 94 °C for 30 s and 60 °C for 1 min for annealing and extension and 10 min at 98 °C for reaction termination. The plate was then placed into a Q × 200 droplet reader for data analysis. The ddPCR analysis was performed with Bio-Rad QX Manager software. A Realtime TaqMan PCR analysis was performed using the ABI Prism 7900 Sequence Detection System (Applied Biosystems, Thermo Fisher Scientific, Waltham, MA, USA) with the TaqMan Universal PCR Master Mix and probes specific to human_COL6A2 (assay Hs00242484_m1), human_COL6A3 (assay Hs00915125_m1) and human GAPDH (assay Hs99999905_m1) from Applied Biosystem (Thermo Fisher Scientific, Waltham, MA, USA). Reactions were performed at 50 °C for 2 min and 95 °C for 10 min, followed by 40 cycles at 95 °C for 15 sec and 60 °C for 1 min. The relative expression of the target genes was normalized to the level of the GAPDH housekeeping gene in the same cDNA by using the 2-ΔΔCT quantification. The replicated Relative Quantity (RQ) values for each biological sample were averaged.

### 2.6. Immunofluorescence and Immunoelectron Microscopy

The primary fibroblasts were seeded on coverslips in wells of a 24-well plate, grown to confluence and treated for 24 h with 0.25 mM of L-ascorbic acid phosphate magnesium (Wako Chemicals GmbH, Neuss, Germany o VWR, Radnor, PA, USA) to induce collage VI secretion. Cells were permeabilized with 0.3% TRITON-X100 and stained with mouse monoclonal anti-collagen VI (clone 3C4) primary antibodies (MAB1944, Merck, Kenilworth, NJ, USA) coupled with an AF488-conjugated anti-mouse secondary antibody (Thermo Fisher Scientific, Waltham, MA, USA) or polyclonal Fitzgerald (Biosynth, Gardner, MA, USA) coupled with a TRITC-conjugated anti-rabbit secondary antibody (DAKO). The collagen I primary antibody (Abcam, Cambridge, UK) was used for collagen I expression. Melanocytes were incubated with a rabbit polyclonal anti-NG2 proteoglycan and a mouse monoclonal anti-collagen VI (clone 3C4) primary antibodies (Merck, Kenilworth, NJ, USA). Cell nuclei were stained with 6-diamidino-2-phenylindole DAPI (1:40,000). Immunofluorescence were visualized using a Zeiss Axioskop 40 FL fluorescence microscope with a digital camera, AxioCam, and AxioVisionRel version 4.8 software for image processing (Carl Zeiss, Milan, Italy) or a Nikon epifluorescence microscope. The quantification of collagen VI fluorescence signals was analyzed using the NIS Elements AR from Nikon. For each sample, ten fields acquired at 100× were analyzed. Only areas with filamentous arrangements, corresponding to the secreted assembled protein, were evaluated, while perinuclear areas with a diffuse pattern, corresponding to the intracellular not secreted chains, were excluded from the analysis. A square of 50 × 50 pixels and the same threshold were set for all images. For rotary shadowing electron microscopy analysis, fibroblasts were incubated with an anti-ColVI antibody diluted 1:25 in a culture medium at 37 °C for 2 h. After several washes with the culture medium, samples were incubated with an anti-mouse IgG 5 nm gold-conjugated antibody diluted 1:20 in the culture medium for 1 h at 37 °C. Negative controls were performed in the absence of the primary antibody. After immunolabeling, cells were fixed with 2.5% glutaraldehyde in a 0.1 M cacodylate buffer and 1% osmium tetroxide, dehydrated in ethanol and critical point dried. Thereafter, the slides were rotary-shadowed with platinum at 45 °C and coated with carbon at 90 °C in a Balzers BAF 400D Freeze Fracture as previously described (Zhang et al., 2002 [29]).

### 2.7. Statistical Analysis

Data were analyzed for statistical significance using a two-way ANOVA or Student’s *t*-test. All values in each group were expressed as mean ± SD. A quantitative analysis of mean fluorescence intensity of ColVI/NG2 ratio was performed on three independent experiments ± standard deviation. Statistically significant differences (*p* < 0.001) were calculated by a Student’s *t*-test. All group comparisons were considered significant at *p* < 0.05, *p* < 0.01, *p* < 0.001.

## 3. Results

### 3.1. Screening of gRNAs for Efficient and Specific Editing of the Mutated COL6A1 Sequence

**Figure 1:**
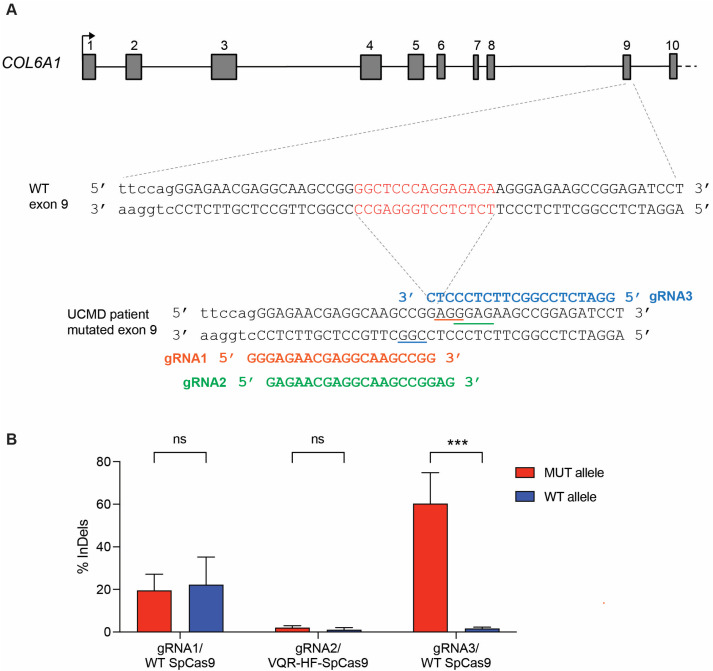
CRISPR/Cas9 allele-specific targeting of c.824_838del COL6A1 dominant variant. (**A**) Schematic representation (not in scale) of the first 10 translated exons of COL6A1 gene Each exon is numbered from 1 to 10. The arrow above the first exon indicates the transcription start site. The sequence of the genomic region surrounding exon 9 in the wild-type (WT) allele and UCMD allele carrying the heterozygous c.824_838del variant are shown (exon 9 in capital letters, the last nucleotides of intron 8 in lower case). The 15-nucleotide deletion is highlighted in red on the WT sequence. The picture illustrates gRNA1 (red), gRNA2 (green) and gRNA3 (light blue) targeting the mutation. PAM sequences are underlined. (**B**) Frequency of indels determined by TIDE analysis on HEK 293T cells transfected with effector plasmids for SpCas9 variants and gRNA and plasmids carrying the WT or UCMD allele sequences. The experiment was performed in triplicate and is presented as mean ± SEM. *** *p* < 0.001; ns, not significant.

To specifically target the heterozygous c.824_838del (NM_001848.2) pathogenic variant in exon 9 of *COL6A1* that leads to the p.Gly275_Lys280del collagen VI variant, three gRNAs for two SpCas9 variants, WT and VQR-HF1, that are high fidelity were designed (Figure 1A).

Two gRNAs, gRNA1 and gRNA2, were aligned to the exon 9 just upstream of the 15-nt deletion and, upon co-expression of WT SpCas9 or the high-fidelity VQRHF1-SpCas9 variant, respectively, the corresponding PAM sequences (5′-AGG-3′ for WT SpCas9, 5′-GGAG-3′ for VQRHF1-SpCas9) immediately downstream of the deletion were engaged (Figure 1A). On the reverse complementary strand, gRNA3 guided WT SpCas9 to the 5′-CGG-3′ PAM sequence and aligned to the exon 9 downstream of the 15-nt deletion (Figure 1A). In the absence of a human cell line carrying the pathogenic variant for gRNA screening, two template plasmids carrying the WT or mutant target region of the *COL6A1* gene were transfected together with CRISPR/Cas9 effector plasmids in HEK293T cells to determine the most efficient gRNA specific for the pathogenic variant. A TIDE analysis on cells transfected with effector plasmids showed a modest indels frequency for gRNA1 on both the mutant and WT sequences but no indels in any templates for gRNA2 (Figure 1B). On the contrary, gRNA3 designed on the reverse strand and annealing downstream on the mutation, scored a 60.33% editing of the mutant template without affecting the WT sequence in three transfection experiments (Figure 1B). Thus, this is an efficient and specific targeting of the c.824_838del pathogenic variant, dictating its use for UCMD fibroblasts correction.

### 3.2. Mutation-Specific Editing in UCMD Fibroblasts Leads to Efficient Frameshift Alteration of the Mutated Allele

**Figure 2:**
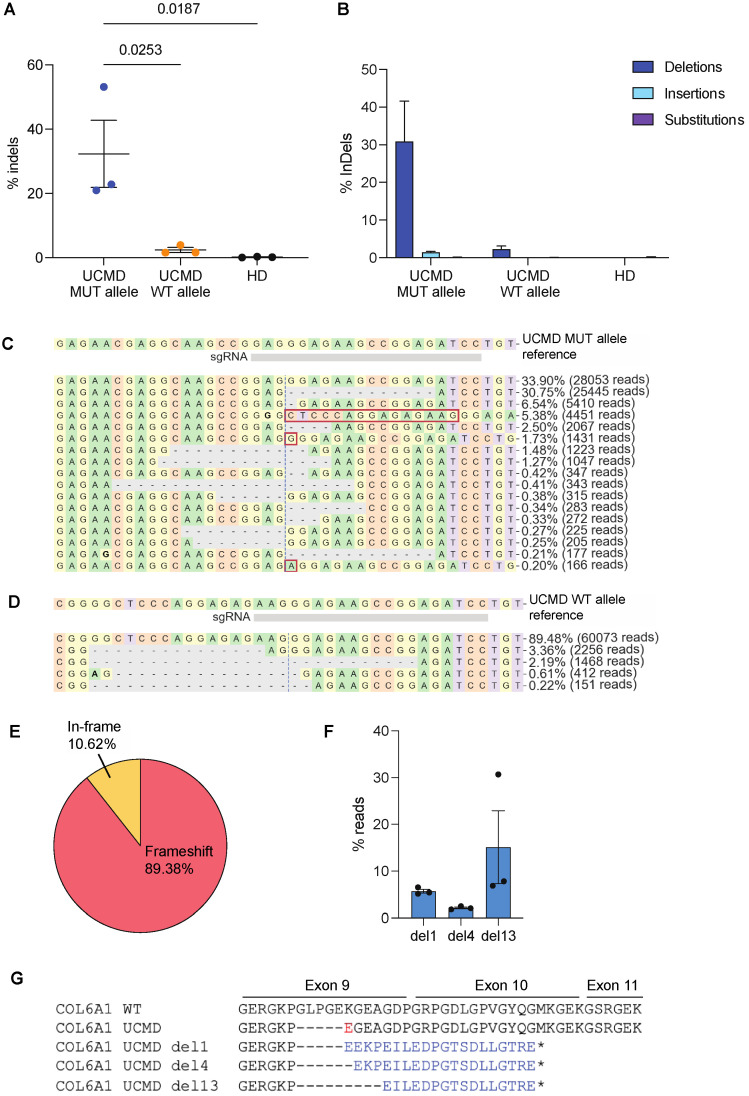
NGS data analysis of UCMD patient cells electroporated with RNP/gRNA3. (**A**) Frequency of indels in exon 9 of WT or mutant allele (MUT) from UCMD fibroblasts or from HD fibroblasts. The experiment was performed in triplicate and is presented as mean ± SEM. * *p* < 0.05. (**B**) Type of indels, and their relative percentage, generated in WT or mutant allele (MUT) from UCMD fibroblasts or from HD fibroblasts. (**C**,**D**) CRISPResso2 graphic representation of the distribution of identified alleles around the cleavage site of the gRNA3 on UCMD mutated allele or on WT allele. The top sequence is the unmodified reference. Substitutions are shown in bold font. Red rectangles highlight inserted sequences. Horizontal dashed lines indicate deleted sequences. The vertical dashed line indicates the predicted cleavage site. A representative experiment is shown. (**E**) CRISPResso2 analysis of indels generated on UCMD MUT allele leading to frameshift or in-frame mutations. The mean of triplicates is presented in pie chart. (**F**) Percentages of the three most frequent mutants (del1, del4, del13) identified by CRISPResso2 upon editing the MUT allele of UCMD fibroblasts. (**G**) Amino acid sequence of exon 9-11 in COL6A1 WT, UCMD c.824_838del variant diagnosed in patient cells and UCMD variants (del1, del4 and del13) identified by CRISPResso2 upon editing the MUT allele of UCMD fibroblasts. The amino acid substitution resulted from the 15-nt deletion in UCMD MUT allele is highlighted in red, while different amino acid sequences resulting from CRISPR-induced frameshift are in blue. Dash indicates deleted codons, and star indicates premature stop codon downstream the canonical one.

To transfer the CRISPR editing tools to patient’s fibroblasts, ribonucleoparticles (RNP) formed by high-fidelity (HF) Alt-R SpCas9 complexed to synthetic gRNA3 or control gRNA were introduced into the patient’s skin fibroblasts by electroporation. In parallel, healthy donor (HD)-derived primary fibroblasts were treated with RNP-gRNA3 or control to assess the potential editing of WT *COL6A1*. Targeted NGS sequencing of three independent experiments were performed on the treated fibroblasts from HD or UCMD patients, and the editing on UCMD WT or c.824_838del alleles was separately analyzed by taking advantage of a SNP (C > T) 62-nt downstream at the end of exon 9. The allele-specific analysis was performed with the CRISPResso2 webtool and showed a 32.31% efficiency of editing the mutated allele and a 2.37% editing of the WT allele, confirming the HF Alt-R SpCas9/gRNA3 complex as the most specific and efficient for the c.824_838del pathogenic variant of COL6A1 (Figure 2A and Figure S1). The analysis on HD fibroblasts treated with RNP-gRNA3 showed barely detectable editing (0.20%), confirming that the WT *COL6A1* allele is not disrupted by gRNA3 (Figure 2A). The most frequent indels occurring in the mutant allele were deletions up to 24 nt (30.89%), while insertion represented 1.45% of editing events (Figure 2B–D). The insertion of 15 nt, resulting in the restoration of the WT *COL6A1* sequence was not detected excluding possible homology-directed repair of the mutated allele upon CRISPR treatment. Of note, despite the differences in the type of indels and their distribution, 89.38% of all edited sequences led to frameshift alterations (Figure 2E and Figure S2). In all replicates, the most frequent CRISPR-induced variants were deletions of 1, 4, 13 nucleotides in exon 9, accounting for 5.70%, 2.11%, and 15.12%, respectively (Figure 2F). Interestingly, these frameshift mutants resulted in premature stop codon in exon 10 (Figure 2G), suggesting a potentially favorable outcome of knocking down the p.Gly275_Lys280del COL6A1 variant. No impact on the splice sites in the *COL6A1* exon 9 of UCMD fibroblasts treated with RNP-gRNA3 was measured by CRISPResso2 (Figure S3).

The major drawback of the CRISPR/Cas9 system is the possibility to induce genome-wide unwanted off-target effects. The COSMID webtool [27] predicted 25 putative genome-wide off-targets (OT) for gRNA3 displaying up to three mismatches and a risk score from 46 to the top-ranked 0.65 value. We prioritized the 14 top ranked OTs with scores less than 10 mapped into a gene or transcription regulatory regions (enhancer, promoter) for a genomic analysis with targeted NGS sequencing. The CRISPResso2 analysis showed barely detectable indels in the predicted OTs (Table 1 and Figure S4). Interestingly, *COL6A3* was one of the potential target sites (OT6), but no editing was detected in the treated fibroblasts.

### 3.3. Allele-Specific Editing Results in Reduced Expression of the COL6A1 Mutated Transcript

**Figure 3:**
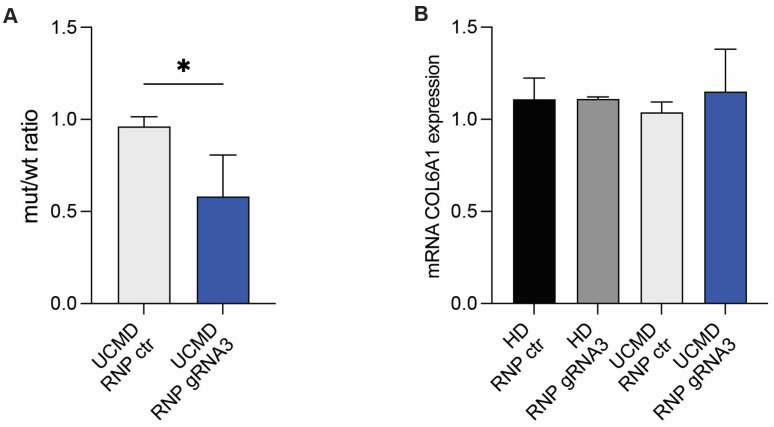
Efficient knockdown of mutant allele expression. (**A**) Measurement of the WT and mutant COL6A1 transcripts by allele-specific ddPCR in UCMD fibroblasts treated with RNP-gRNA3 or control RNP (RNP ctr). Mutant/WT COL6A1 allele ratio in UCMD patient fibroblasts treated with RNPs is reported in the column chart. Mean ± SD is shown. *, *p* < 0.05. (**B**) Measurement of mRNA levels of a total COL6A1 expression by ddPCR in HD and UCMD fibroblasts treated with control RNP (RNP ctr) or RNP-gRNA3. The results of four experiments are shown as mean ± SD.

To verify that the CRISPR-mediated targeting of the c.824_838del variant resulted in the specific downregulation of the expression of the mutant allele, we analyzed the expression of *COL6A1* transcripts in patient-derived fibroblasts electroporated with mutation-specific RNP-gRNA3 or control RNP. The ddPCR analysis with probes designed to distinguish mutant and WT *COL6A1* transcripts showed a significantly lower ratio between the mutant transcript over the WT one, compared to the control UCMD fibroblasts (ratio: RNP ctr = 0.96, RNP-gRNA3 = 0.58, *p* = 0.0136, Figure 3A), calculated in five independent experiments. The ddPCR analysis showed that the overall level of *COL6A1* transcripts was not affected by the treatment with RNP-gRNA3, compared to control RNP, both in HD and the patient’s fibroblasts (Figure 3B). As a control, the expression of mRNA of *COL6A2* and *COL6A3* were evaluated by an RTqPCR in HD and UCMD fibroblasts treated with RNPs. The CRISPR editing specific for *COL6A1* did not affect the expression of the *COL6A2* and *COL6A3* chains—a relevant issue since an intron of the *COL6A3* gene was scored as a putative off-target site (Figure S5A,B).

### 3.4. CRISPR-Mediated Editing Tailored to the Mutated COL6A1 Allele Restores α1 Chain of Collagen VI Protein and Structure in the Extracellular Matrix

**Figure 4:**
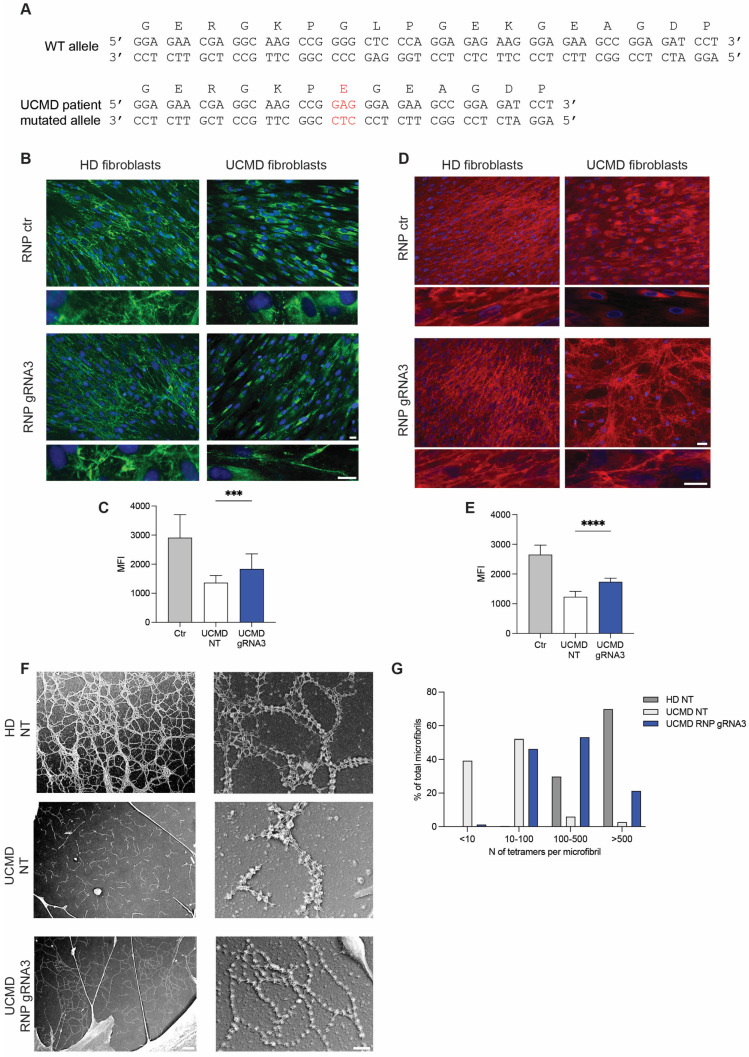
Secretion of collagen VI in fibroblasts from healthy donor (HD) and UCMD patient after treatment with RNP. (**A**) Nucleotide and amino acid sequence of exon 9 in UCMD patient carrying c.824_838del variant. In red, the insertion of a Glu residue is shown. (**B**) Immunofluorescence analysis with antibody specific for collagen VI (MAB1944) of fibroblasts from HD or UCMD patient, treated with control RNP (RNP ctr) or RNP-gRNA3. The experiment was performed in quadruplicate. A representative experiment is shown. Enlarged image insets show the structure of collagen VI microfibrils. Scale bars indicate 20 µm magnification. (**C**) Mean intensity of collagen VI quantified in fibroblasts treated with RNP-gRNA3. Data represent mean ± SD from analysis of 10 individual field images acquired at 100× original magnification under fluorescence microscopy. *** *p* < 0.001). (**D**) Immunofluorescence analysis with collagen VI-specific antibody (polyclonal Fitzgerald) of primary fibroblasts from HD or UCMD patient treated with control RNP (RNP ctr) or RNP-gRNA3. The polyclonal Fitzgerald anti-collagen VI antibody was used to label *α*1, *α*2 and *α*3 chains of collagen VI. A representative experiment is shown. Enlarged image insets show the structure of collagen VI microfibrils. Scale bars indicate 20 µm magnification. (**E**) Mean intensity of collagen VI quantified in fibroblasts treated with RNP-gRNA3. Data represent mean ± SD from analysis of 10 individual field images acquired at 100× original magnification under fluorescence microscopy. **** *p* < 0.0001). (**F**) Ultrastructural analysis by rotary-shadowing electron microscopy of collagen VI microfibrillar network in untreated (NT) HD fibroblasts, UCMD fibroblasts treated with RNP-gRNA3 or not (UCMD NT). (**G**) Percentage of microfibrils composed by increasing amounts of tetramers in UCMD fibroblasts treated with RNP-gRNA3 or not (UCMD NT) and in control fibroblasts (HD NT).

The heterozygous c.824_838del mutation identified in the UCMD patient caused the loss of six amino acid residues (GlyLeuProGlyGluLys), and the insertion of a Glu missense residue resulted from the fusion codon GAG in the TH domain of an α1 chain of collagen VI (Figure 4A). Although the coding frame is conserved, the deletion of these residues compromises the extracellular microfibril assembly and leads to a dramatic reduction of the secretion of collagen VI in skin fibroblasts (Figure 4B,C) as observed in muscle cells [20]. The UCMD patient fibroblasts treated with RNP-gRNA3 or RNP-ctr were incubated with 0.25 mM ascorbic acid for 1–5 days to trigger collagen VI release in the ECM. The immunofluorescence analysis demonstrated a marked increase in collagen VI deposition in the ECM in UCMD fibroblasts treated with RNP-gRNA3 compared to fibroblasts treated with RNP-ctr. Interestingly, staining with antibodies specific for either the α3 chain or assembled collagen VI chains further supported the folding of mature collagen VI (Figure 4B–E). Collagen VI-positive microfibrils in HD fibroblasts treated either with RNP-ctr or RNP-gRNA3 were comparable to untreated cells, indicating that the electroporation of RNP did not affect collagen VI secretion. CRISPR-mediated disruption of the mutant allele induced not only an increase in collagen VI secretion in ECM but also an improvement in microfibril structure. Indeed, the ultrastructural analysis by rotary-shadowing electron microscopy of collagen VI performed on untreated UCMD fibroblasts showed short collagen VI microfilaments, which formed a poor extended and disorganized network. Interestingly, after the RNP-gRNA3 treatment, the pattern of the collagen VI network was markedly improved together with a relevant restoration of the regularly beaded microfibril network (Figure 4F). The ability of the tetramers to associate end-to-end in the extracellular matrix of the UCMD patient fibroblasts before and after CRISPR treatment, compared to the HD microfilament, was quantified. In the untreated UCMD culture, about 53% of microfibrils were constituted by 10 to 100 tetramers and 5% by up to 500 tetramers, and only 2% of the microfibrils contained more than 500 tetramers. After CRISPR treatment, about 55% of fibrils were constituted by 100 to 500 tetramers, and 20% were formed by more than 500 tetramers (Figure 4G), indicating that tetramer–tetramer association appears markedly improved.

The treatment of UCMD or HD fibroblasts with RNP-gRNA3 did not affect the expression of collagen I and fibronectin (Figure S6), demonstrating that CRISPR editing of collagen VI does not interfere with the production and assembly of other proteins of the extracellular matrix.

### 3.5. Restored Collagen VI-NG2 Axis in Melanocytes

**Figure 5:**
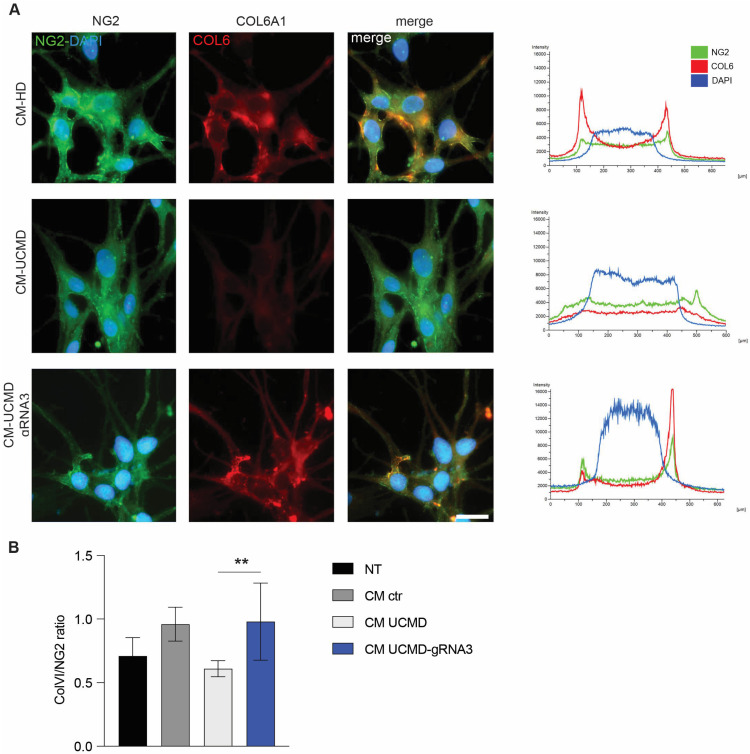
Restoration of binding of NG2 by collagen VI secreted by UCMD patient after treatment with RNP. (**A**) Immunofluorescence analysis of NG2 and collagen VI in melanocytes from healthy donor treated with collagen VI-containing conditioned medium (CM) of fibroblasts from HD or UCMD patient untreated or treated with RNP-gRNA3. The experiment was performed in triplicate. Representative mean fluorescence intensity profiles of each sample are shown on the right of each row. A representative experiment is shown. Scale bar indicates 20 µm magnification. (**B**) COL6/NG2 ratio in melanocytes untreated (NT) or cultivated with conditioned medium (CM) of fibroblasts from HD (ctr), UCMD or UCMD treated with RNP-gRNA3. The experiment was performed in triplicate, and the mean ± SEM is shown. ** *p* < 0.01.

Mutated collagen VI chains lose the ability to bind specific cell receptors, as the NG2 proteoglycan, and the ECM surrounding the cells, destroying the cytoskeletal-ECM axis with dramatic consequences on critical cellular functions. To explore whether the collagen VI protein secreted by CRISPR-treated fibroblasts recovered its binding properties, we cultured fibroblasts from HD, UCMD and CRISPR-corrected UCMD in the presence of ascorbic acid, and the conditioned medium (CM) was collected for an in vitro binding assay. Of note, the bulk population of CRISPR-corrected fibroblasts and controls was cultured for a week before raising the conditioned medium. Since normal melanocytes, similar to muscle cells, express the NG2 proteoglycan in culture but do not synthesize collagen VI (Figure S7), they were used to evaluate the binding properties of collagen VI secreted in the CM from UCMD-untreated or CRISPR-treated compared to HD cells. As shown in Figure 5A, collagen VI from CRISPR-treated cells, as compared to UCMD-untreated, showed a clear association with the NG2 receptor on the cell surface, as indicated by the overlap of both fluorescent signals. Moreover, the collagen VI/NG2 ratio in melanocytes cultivated with CM from CRISPR-corrected UCMD fibroblasts is comparable to that measured in melanocytes cultivated with CM from HD fibroblasts and significantly different from those cultivated with CM from UCMD fibroblasts (*p* = 0.003) or without any CM (Figure 5B). Altogether, these data indicate that the collagen VI secreted by CRISPR-corrected UCMD fibroblasts recovers the ability to anchor the cell surface, restoring potentially the cytoskeletal-ECM axis.

## 4. Discussion

The most common types of mutations affecting *COL6A* genes and diagnosed in UCMD patients are missense and in-frame mutations. Among the in-frame variants, the loss of approximately five amino acids accounts for 70% of the deletions in COL6A coding regions [19]. In 2003, a seminal study by Pan et al. [30] described a patient carrying a de novo heterozygous in-frame deletion in the TH domain of *COL6A1* mRNA (c.805-903del) that excluded exon 9 and 10 but maintained the cysteine residue at position 89 of the TH, which is important for dimer assembly. This mutation resulted in a severe phenotype of classical UCMD, thus demonstrating that UCMD can be caused by dominantly acting mutations. Since that time, it has been observed that the majority of patients, including many with the most severe phenotype, carry dominant negative pathogenic variants [31], which can be tackled, as a therapeutic strategy, by silencing the mutant allele.

Here, we report a CRISPR/Cas9-mediated approach for a heterozygous dominant p.Gly275_Lys280del in-frame variant mapped in exon 9 of the *COL6A1* gene in the UCMD patient who never achieved the ability to stand and walk. This mutation resulted in a marked reduction of collagen VI and in mitochondrial dysfunction in the patient’s muscle cultures [20].

The CRISPR strategy developed to knock out the p.Gly275_Lys280del variants in the patient’s fibroblasts provided more than 32% editing of the mutant allele and less than 0.2% editing in the HD fibroblasts. The vast majority of the edited sequences (89%) led to frameshift and resulted in a significant downregulation of the mutated transcript, keeping unaltered the overall levels of *COL6A1*, *COL6A2* and *COL6A3* transcripts. The expression of high-fidelity Alt-R SpCas9 combined to a single gRNA into RNP guaranteed negligible levels of off-target editing, as confirmed by the NGS analysis. Of note, the expression of *COL6A3*, one of the predicted off-targets, was not affected by Cas9 treatment, indicating that the transient expression of the CRISPR system provided by RNP resulted in the absence of genotoxic or cytotoxic effects in the treated patient’s fibroblasts.

The p.Gly275_Lys280del mutation leads to the loss of six amino acid residues (GlyLeuProGlyGluLys) and the gain of a Glu missense residue in the TH domain of an α1 chain of collagen VI. The lack of two Gly-X-Y amino acid triple repeats and the presence of an additional negative charged residue in the TH region account for short collagen VI microfilaments, with less than 100 tetramers per microfibril, and a disorganized network. The CRISPR treatment of patient fibroblasts heavily defective for collagen VI secretion allowed the formation of long branched collagen VI polymers, an improved tetramer–tetramer association of the collagen VI fibers, and a significant increase in collagen VI deposition in ECM, supporting the efficient recovery in fibroblasts and showing a marked reduction of collagen VI secretion at the baseline [17].

Moreover, we investigated for the first time the binding of collagen VI secreted by CRISPR-treated fibroblasts to the NG2 proteoglycan receptor. It has been reported that the collagen VI-NG2 axis is disrupted in muscle [32] and tendon fibroblasts in UCMD patients affected by different ColVI mutations [7,33], including a heterozygous mutation exon8-intron 8 c.798_804+8del 15 in the *COL6A1* gene (p.Pro254_Glu268 del), which removed the exon8-intron8 junction causing variable splicing phenotypes, consisting of exon skipping, intron retention and cryptic splice site activation/usage [34,35]. This mutation in the UCMD tendon fibroblasts caused a significant reduction of the NG2 protein as a result of a post-transcriptional regulatory mechanism affected by the presence of a collagen VI mutation. The disruption of the collagen VI-NG2 axis destabilized cell polarization during migration. Since cultured normal melanocytes express high levels of NG2 but not collagen VI, we cultured HD melanocytes with a conditioned medium from CRISPR-treated UCMD fibroblasts, showing that the collagen VI secreted by a bulk population of the patient’s fibroblasts colocalizes with the NG2 proteoglycan receptor expressed in melanocytes, indicating the ability to anchor the cell surface, thus potentially restoring the cytoskeletal-ECM axis. A proper link between muscle cells and the ECM, guaranteed by collagen VI microfibrils, is required to maintain skeletal muscle function and integrity in vivo [2,36].

One of the major unsolved issues related to this strategy is whether the already deposited mutant collagen VI would continue to exert its effect on the non-mutated or corrected collagen VI. Notably, the CRISPR strategy employed in this study resulted in almost complete abrogation of the collagen VI variant in the patient’s fibroblasts as demonstrated by approximately the 90% frameshift of edited alleles resulting in premature termination in exon 10 of the *COL6A1* gene. Since the patient’s fibroblasts that are heterozygous in the p.Gly275_Lys280del variant show a heavy impairment in collagen VI secretion and retain the collagen VI complex in the intracellular compartment, the restoration of functional collagen VI upon CRISPR correction suggests that the mutated protein is progressively degraded and not synthetized anymore. To support this evidence, Castroflorio et al. [37] showed that the CRISPR-mediated correction of missense mutation p.G293R in patient-derived fibroblasts resulted in the recovery of the collagen VI extracellular network. Moreover, in this study, we demonstrated the binding of collagen VI upon CRISPR treatment to the NG2 proteoglycan receptor on melanocytes as a consequence of a reduced dominant negative effect on the WT *α*1 chain and degradation of mutated collagen VI.

A variety of CRISPR strategies, which include HDR-mediated repair or a mutation specific knockout of dominant missense mutations [17,18] and the introduction of two DSB to remove a deep intronic variant [16], have been proposed so far for UCMD, reporting the improvement of collagen VI secretion in patients’ fibroblasts. Since the HDR resulted in a poor correction of a missense variant [17], an allele specific gRNA combined to SpCas9 nuclease has been the strategy of choice to tackle the dominant mutations. Our study supports the relevance of this strategy and expands its application to the in-frame deletion variants in collagen genes. Although CRISPR base editing might be proposed in the near future for missense mutations, in-frame deletion/insertion still requires allele-specific knockout or CRISPR prime editing to be able to re-write a short DNA fragment [38]. This engineered potent CRISPR tool suffers from a cumbersome design/optimization of pegRNA and a still limited application in vivo due to delivery methods, which to date rely on AAV vectors or only recently, mRNA packaged into lipid nanoparticles (LNP) [39]. In our study, we employed RNP for in vitro editing to mimic a possible translational application of CRISPR for UCMD.

Indeed, to envisage a clinical translation of the CRISPR approach for UCMD, the delivery of CRISPR components to target cells is still a crucial issue to solve. Gold nanoparticles, LNP and vector-like particles have been proposed for many in vivo gene therapy interventions based on CRISPR systems with either nuclease, base editors or prime editors [40]. While multiple clinical trials have successfully targeted the liver in vivo [41,42], no active or recruiting trials for muscle dystrophies have been submitted so far.

## 5. Conclusions

Our study demonstrates that by knocking out the mutant *COL6A1* allele, UCMD fibroblasts treated with CRISPR/Cas9 secreted collagen VI that correctly assembles in the extracellular matrix. In addition, we demonstrated in vitro that collagen VI secreted by edited UCMD fibroblasts regained its structural and binding properties, suggesting a beneficial effect on skeletal muscle fibers.

## Figures and Tables

**Table 1:** Indels analysis of off-target sites predicted for gRNA3 with COSMID webtool. OT, off-target site; Chr, chromosome; % indels represents the frequency of indels detected by TIDE analysis with *p* < 0.05. Red letters indicate mismatches between gRNA3 sequence 5′>3′ and genomic locus indicated in the Chr position column. Blue letter indicates a nucleotide insertion.

OT	Sequence 5′>3′	COSMID Score	Chr Position	Locus	Element	% Indels
1	GGAgCcCaGGCTTCTCCCTC	0.65	Chr22:50545027-50545049	N.A.	Promoter/ Enhancer	0.271
2	GGAgCTgCtGCTTCTCCCTC	0.75	Chr22:40023977-40023999	FAM38F	Intron	0.204
3	cGcTCTCCGtCTTCTCCCTC	0.77	Chr12:113462617-113462639	LHX5	Exon— 3′UTR	0.105
4	GcAgCTCCGtCTTCTCCCTC	0.8	Chr16:4885192-4885214	PPL	Exon	0.184
5	GGccCTCCGGtTTCTCCCTC	1.02	Chr7:95396673-95396695	PON3	Promoter/ Enhancer	0.232
6	GGA-CTCCaGCTTCTCCCTC	1.03	Chr2:237403198-237403219	COL6A3	Intron— 5′UTR	0.358
7	GGATCtTCCtGCTTCTCCCTC	1.26	Chr2:19118696-19118719	LINC01376	Intron— lncRNA	0.120
8	GGAcCTCCtGCTcCTCCCTC	1.62	Chr14:105571653-105571675	N.A.	Enhancer	0.117
9	GGATCTtCtGCTTCTCaCTC	3.58	Chr6:41643886-41643908	MDFI	Intron	0.308
10	GGATCTCCG-CTTCTCtCTC	3.86	Chr9:37885678-37885699	SLC25A51	Intron	0.289
11	GGATCTCCtGCTTCTCCCgC	5.35	Chr7:1487857-1487879	INTS1	Exon	0.123
12	GGATCTCtGGCTgCTCCCTt	7.37	Chr4:22782246-22782268	GBA3	Intron	0.201
13	GGATCTCtGGCTTgTCCCTt	7.57	Chr2:28361239-28361261	N.A.	Enhancer	0.857
14	GGATgTCCGGCTTCTCCtcC	9.19	Chr21:41379467-41379489	MX2	Intron	2.000

## Data Availability

The data assessed and reported here can be obtained from the authors upon reasonable request following ethical and privacy principles.

## Supplementary Materials

The following supporting information can be downloaded at: https://www.mdpi.com/article/10.3390/biom14111412/s1, Figure S1: CRISPResso2 analysis of UCMD fibroblasts treated with RNP-gRNA3; Figure S2: CRISPResso2 analysis of frameshift alterations; Figure S3: CRISPResso2 analysis of splicing alterations; Figure S4: CRISPResso2 graphic representation of distribution of identified alleles (OT) targeted by gRNA3; Figure S5: Real time TaqMan PCR for COL6A2 and COL6A3 genes; Figure S6: Real time TaqMan PCR for COL6A2 and COL6A3 genes; Figure S6: Immunofluorescence for Collagen I; Figure S7: Immunofluorescence for NG2 and collagen VI; Table S1: Primers used in this study.
