# Supplementary material for: Restored Collagen VI Microfilaments Network in the Extracellular Matrix of CRISPR-Edited Ullrich Congenital Muscular Dystrophy Fibroblasts
Source: Biomolecules. 2024 Nov 6;14(11):1412. doi: 10.3390/biom14111412 (PMC11591638; doi:10.3390/biom14111412)
Supplement: Supplementary file 1 [file biomolecules-14-01412-s001.zip › biomolecules-3242627-supplementary.pdf]

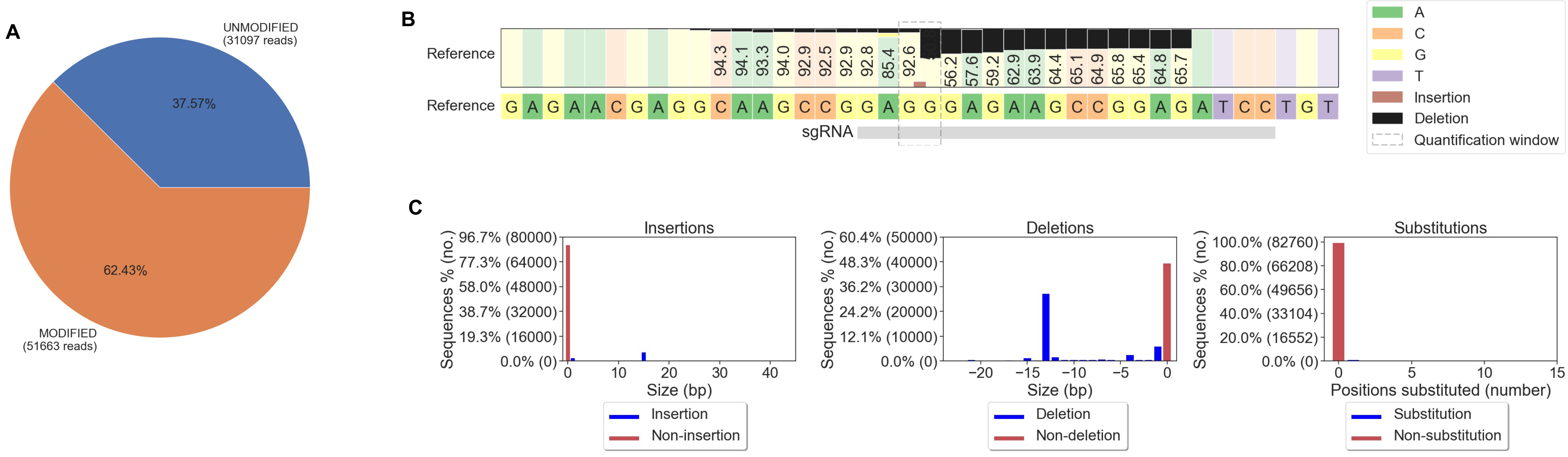

Figure S1. CRISPResso2 analysis of UCMD fibroblasts treated with RNP-gRNA3 A) Frequency and absolute numbers of modified (orange) and unmodified (blue) reads in a representative experiment of targeted NGS sequencing. **B)** Nucleotide distribution around the gRNA3 target site. **C)** Frequency distribution of sequence modifications that increase (insertions, left panel) or reduce (deletions, middle panel) read length with respect to the reference amplicon. Frequency distribution of sequence modifications that do not alter read length with respect to the reference amplicon (substitutions, right panel). A representative replicate is shown.

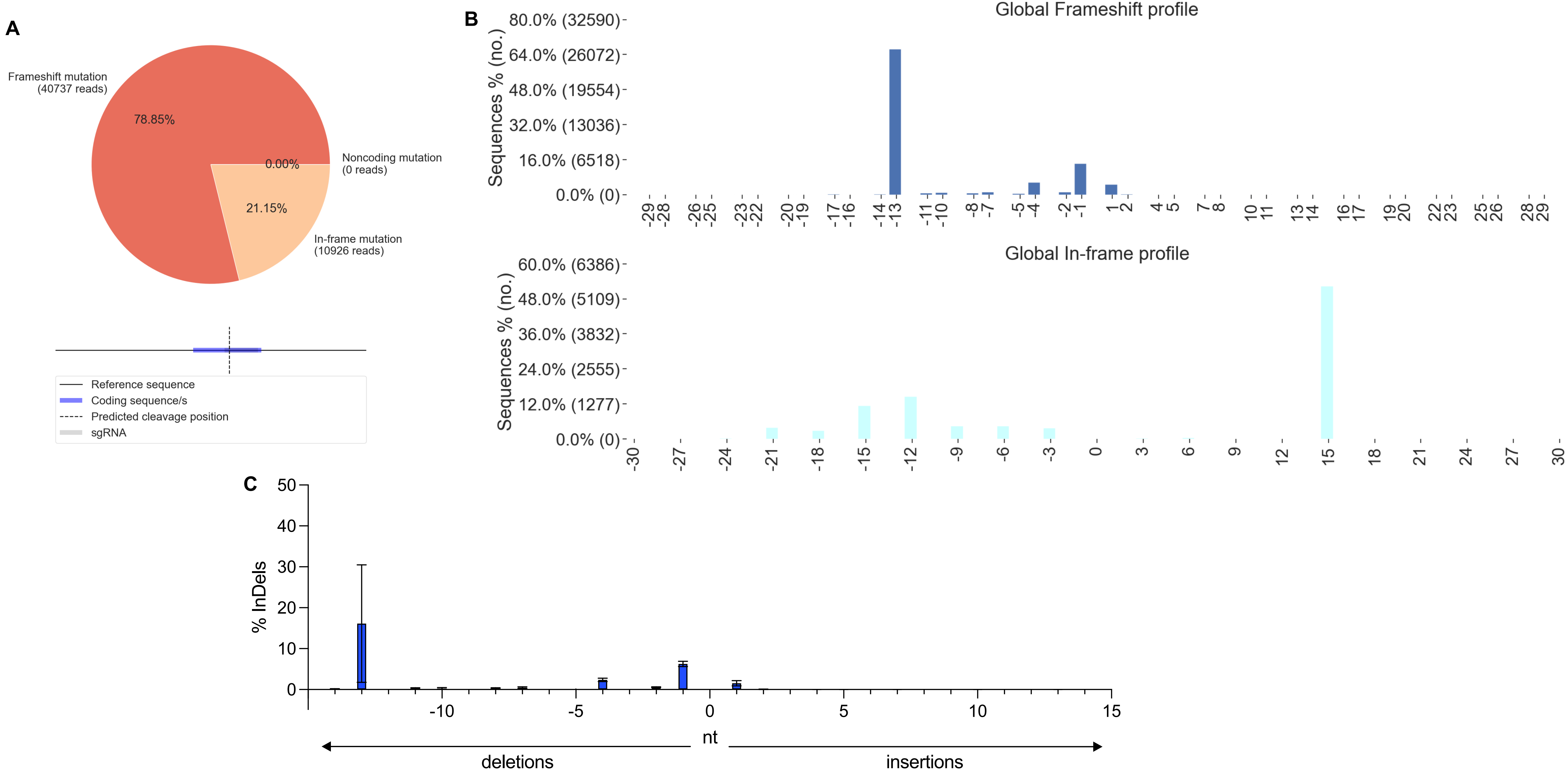

Figure S2. CRISPResso2 analysis of frameshift alteration. A) CRISPResso2 analysis of indels generated by RNP-gRNA3 on UCMD mutant allele leading to frameshift or in frame mutations. A representative experiment is shown. **B)** Frameshift (upper panel) and in-frame (lower panel) mutagenesis profiles in UCMD mutant allele targeted by RNP-gRNA. indicating position affected by modification. Reads with no length modifications are not shown. **C)** Distribution of frameshift mutations in UCMD mutant allele of patient fibroblasts treated with RNP-gRNA3. The results of three experiments are shown as mean ± SD.

A

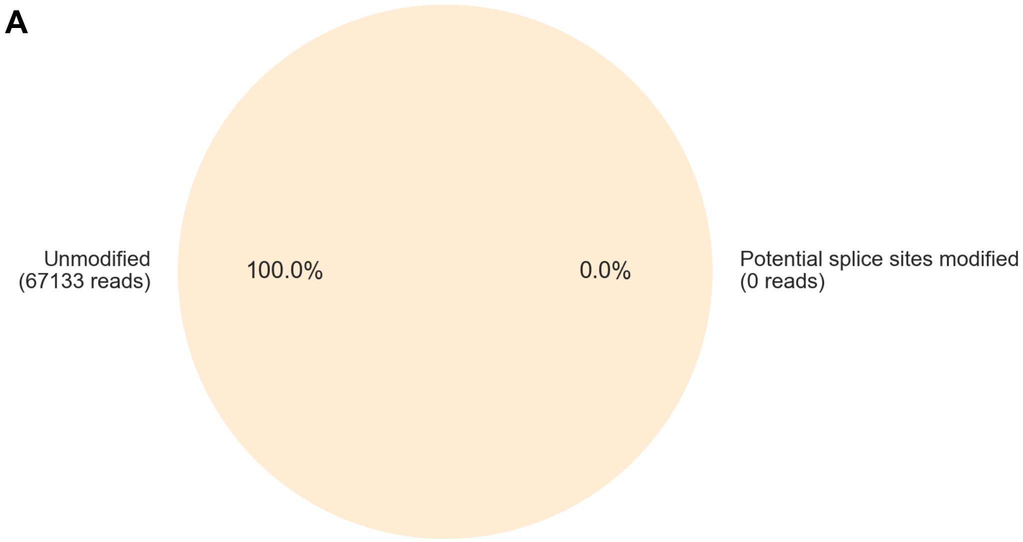

B

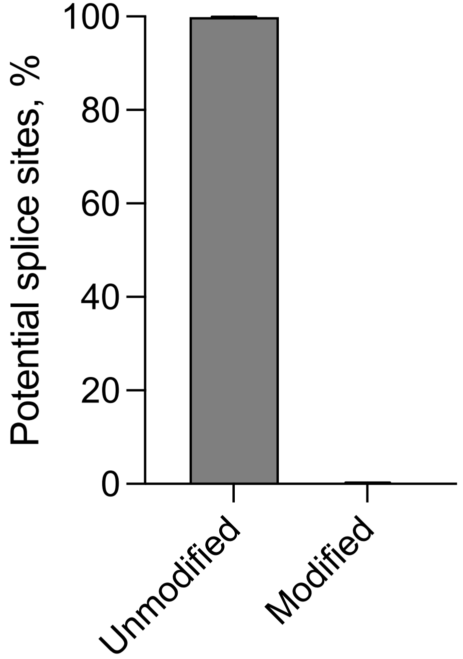

Figure S3. CRISPResso2 analysis of splicing alteration. A) CRISPResso2 analysis of predicted impact on splice sites in COL6A1 exon 9 of UCMD fibroblasts treated with RNP-gRNA3. Potential splice sites modified refers to reads in which the either of the two intronic positions adjacent to exon junctions are disrupted. **B)** Percentage of potential splice sites modified or not in UCMD mutant allele of patient fibroblasts treated with RNP-gRNA3. The results of three experiments are shown as mean ± SD.

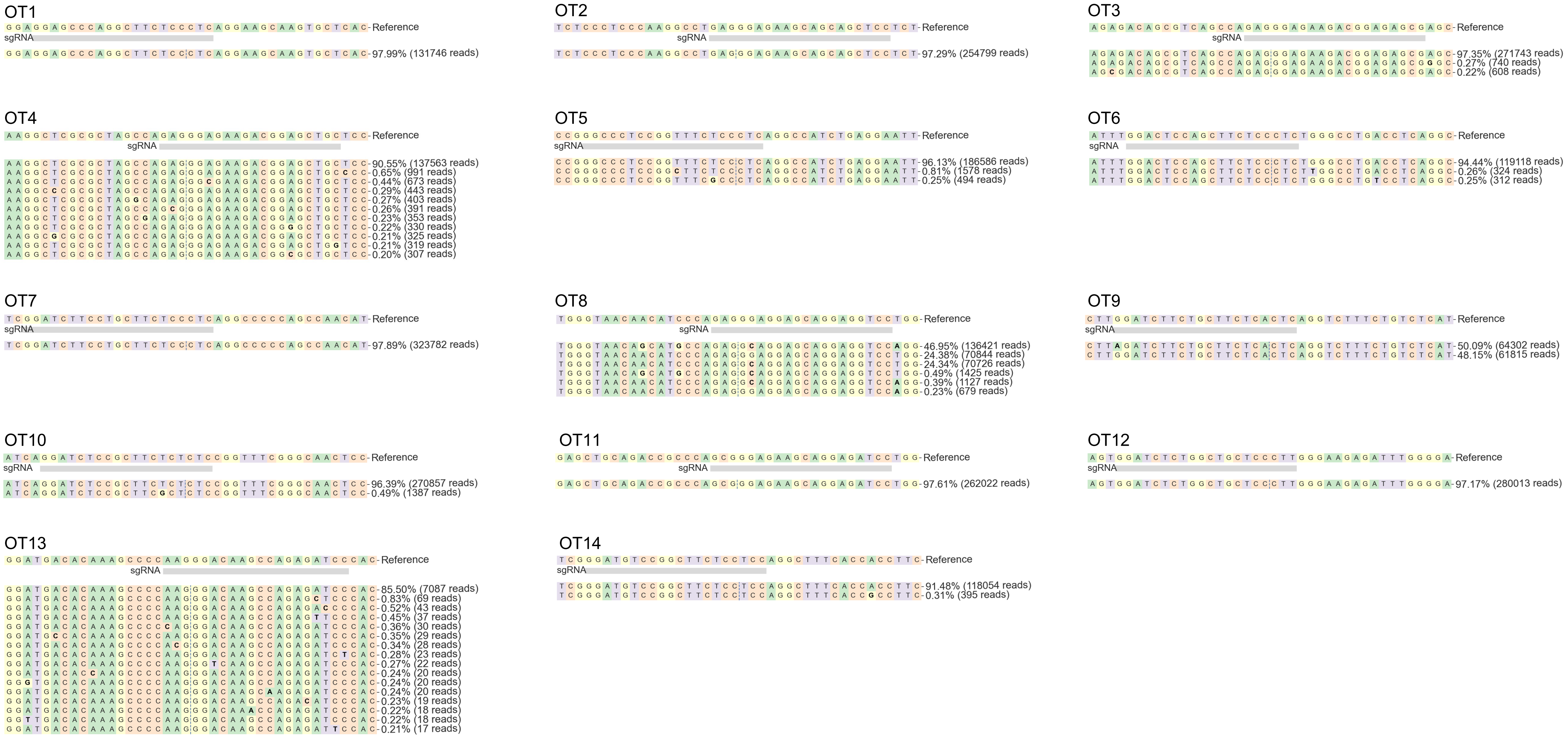

Figure S4. CRISPResso2 graphic representation of distribution of identified alleles (OT) targeted by gRNA3. The top sequence is the unmodified reference. Substitutions are shown in bold font. The vertical dashed line indicates the predicted cleavage site.

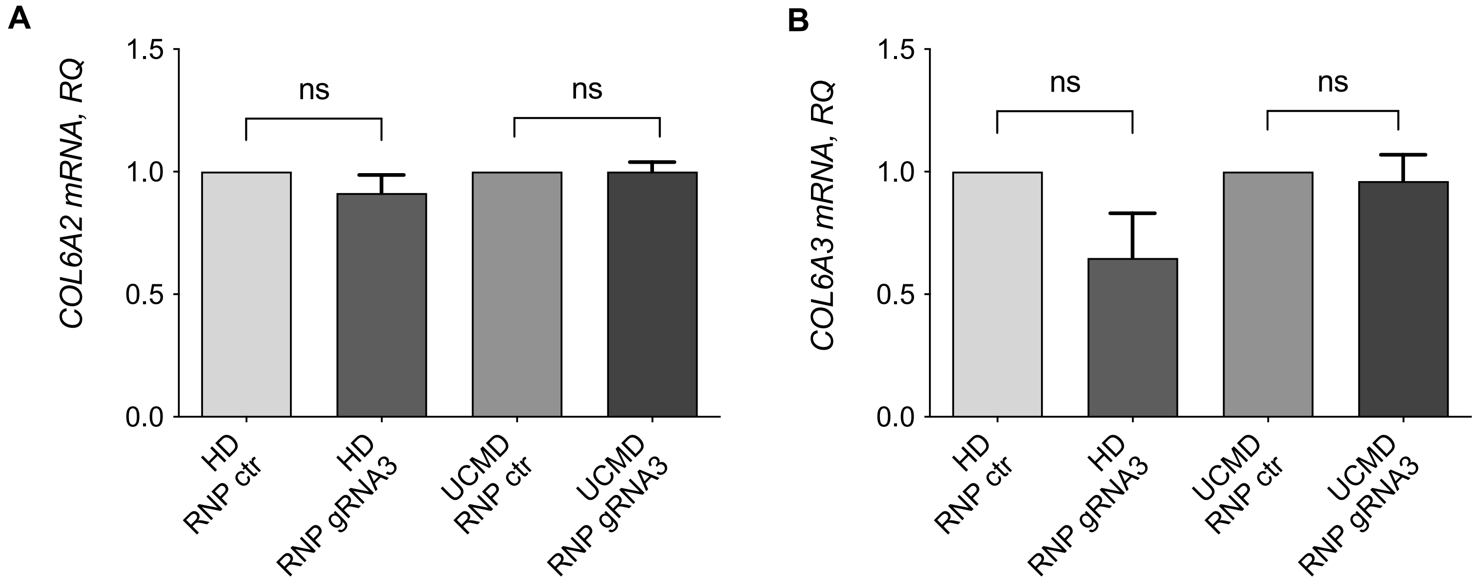

Figure S5. Real time TaqMan PCR on COL6A2 and COL6A3 genes. Real time TaqMan PCR on *COL6A2* **(A)** and *COL6A3* **(B)** expression in fibroblasts from HD or UCMD patient treated with control RNP (RNP ctr) or RNP-gRNA3. The results of 4 experiments are shown as mean ± SD. ns, not significant difference.

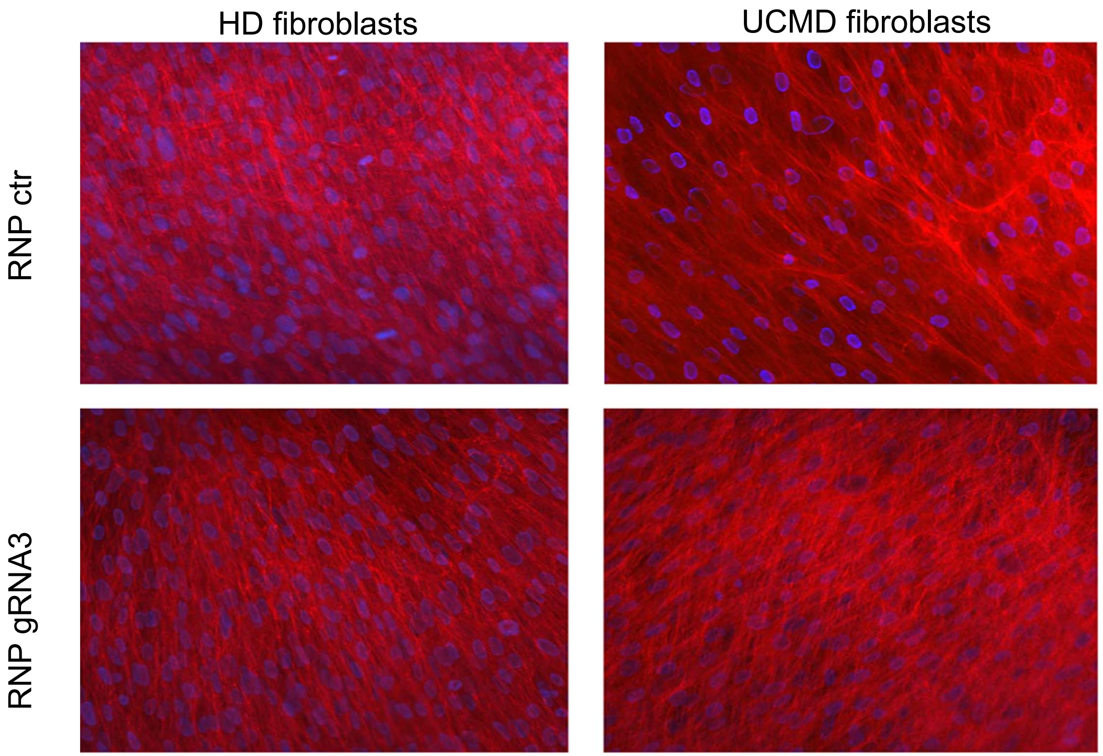

Figure S6. Immunofluorescence for Collagen I. Immunofluorescence analysis for collagen I on primary fibroblasts from HD or UCMD patient treated with control RNP (RNP ctr) or RNP-gRNA3. A representative experiment is shown.

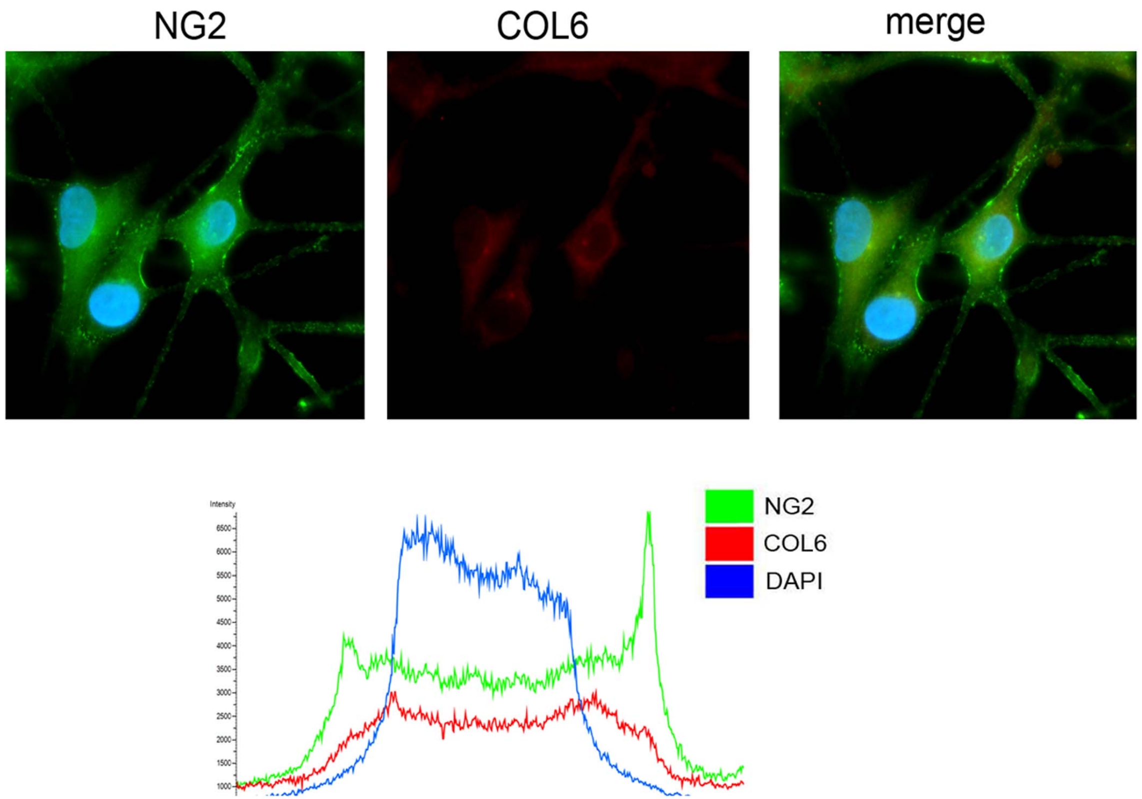

Figure S7. Immunofluorescence analysis for NG2 and collagen VI. Immunofluorescence analysis of untreated melanocytes labelled with anti-NG2 or anti-collagen VI antibodies. The fluorescence intensity profile is shown.

Table S1. Primer used in this study

Primer ID	Primer sequence (5' to 3')	Application
gRNA1_F	CACCGGGAGAACGAGGCAAGCCGG	Oligo annealing
gRNA1_R	AAACCCGGCTTGCCTCGTTCTCCC	Oligo annealing
gRNA2_F	CACCGAGAACGAGGCAAGCCGGAG	Oligo annealing
gRNA2_R	AAACCTCCGGCTTGCCTCGTTCTC	Oligo annealing
gRNA3_F	CACCGAGAACGAGGCAAGCCGGAG	Oligo annealing
gRNA3_R	AAACCTCCGGCTTGCCTCGTTCTC	Oligo annealing
COL6A1_F	AGACAGGAACGAAGGCAGG	PCR
COL6A1_R	GGATAGGATTTGGGCCAAGG	PCR
COL6A1_ddPCR-For	GTGTGCTGCTCCTTCGAATG	ddPCR
COL6A1_ddPCR-Rev	CCTTCATTCCCTGGTACCCA	ddPCR
COL6A1-WT-Probe	[FAM]GGGGCTCCCAGGAGAGAAG[BHQ1]	ddPCR (probe for WT transcript)
COL6A1-MUT-Probe	[HEX]GGCAAGCCGGAGGGAGAAGC[BHQ1]	ddPCR (probe for mutant transcript)
COL6_V2_F	TCGTCGGCAGCGTCAGATGTGTATAAGAGACAGCTTTAAAGCCCTTGATCCCTGAAG	1st PCR amplification
COL6_V3_R	GTCTCGTGGGCTCGGAGATGTGTATAAGAGACAGGGCACTCAGAACCCAGCCAC	1st PCR amplification
OT1_NC_000022_v2_F	TCGTCGGCAGCGTCAGATGTGTATAAGAGACAGAAAAATACACACGGTACTTTCCAGGG	1st PCR amplification
OT1_NC_000022_v2_R	GTCTCGTGGGCTCGGAGATGTGTATAAGAGACAGCTTCCCTTATCTGAGCTGAGGACT	1st PCR amplification
OT2_FAM38_F	TCGTCGGCAGCGTCAGATGTGTATAAGAGACAGCCGTTCCTTGTCCGTCCTTTAG	1st PCR amplification
OT2_FAM38_R	GTCTCGTGGGCTCGGAGATGTGTATAAGAGACAGAGGTTGAGGTTGCAATGAGCTGT	1st PCR amplification
OT3_LXH5_V2_F	TCGTCGGCAGCGTCAGATGTGTATAAGAGACAGAGAGAGATTGAAAGAGAGGGAGAGG[illegible]	1st PCR amplification
OT3_LXH5_V2_R	GTCTCGTGGGCTCGGAGATGTGTATAAGAGACAGCAGCCCTATTCCTAGAGAAGCTGAC[illegible]	1st PCR amplification
OT4_PPL_F	TCGTCGGCAGCGTCAGATGTGTATAAGAGACAGTCCAGGACAAGCTCAAGAGGCTA	1st PCR amplification
OT4_PPL_R	GTCTCGTGGGCTCGGAGATGTGTATAAGAGACAGTCCTGCACCACCACTTTGGC	1st PCR amplification
OT5_PON3_V2_F	TCGTCGGCAGCGTCAGATGTGTATAAGAGACAGTCCTGGAGAAGATGAGTTAAAGCT	1st PCR amplification
OT5_PON3_V2_R	GTCTCGTGGGCTCGGAGATGTGTATAAGAGACAGGAGTTGTGGTGGGAGTTTGCTC	1st PCR amplification
OT6_COL6A3_V2_F	TCGTCGGCAGCGTCAGATGTGTATAAGAGACAGTTCATTCCCTTGTTTGTGGCTTC	1st PCR amplification
OT6_COL6A3_V2_R	GTCTCGTGGGCTCGGAGATGTGTATAAGAGACAGCAGATGGACCACTTAGCAAAGGTAC[illegible]	1st PCR amplification
OT7_LINC01376_V2_F	TCGTCGGCAGCGTCAGATGTGTATAAGAGACAGTGGGCAGACTCTTCCTAACTTGATA	1st PCR amplification
OT7_LINC01376_V2_R	GTCTCGTGGGCTCGGAGATGTGTATAAGAGACAGGAACCAAGACAAGGTGCTTCTCTAA[illegible]	1st PCR amplification
OT8_E1746953enhD_F	TCGTCGGCAGCGTCAGATGTGTATAAGAGACAGCTGTCCTGTTCTCTGACCATCC	1st PCR amplification
OT8_E1746953enhD_V2_F	GTCTCGTGGGCTCGGAGATGTGTATAAGAGACAGAAACCCACAGATACACACGGGA	1st PCR amplification
OT9_MDFI_V2_F	TCGTCGGCAGCGTCAGATGTGTATAAGAGACAGGCATTTTCTTTCCTCCTTGAAACCC	1st PCR amplification
OT9_MDFI_V2_R	GTCTCGTGGGCTCGGAGATGTGTATAAGAGACAGAAGTTGCCATGAAAAGGGAGGCT	1st PCR amplification
OT10_SLC25A51_V2_F	TCGTCGGCAGCGTCAGATGTGTATAAGAGACAGAAGAATTGCGTTGGCCACTTTAC	1st PCR amplification
OT10_SLC25A51_V2_R	GTCTCGTGGGCTCGGAGATGTGTATAAGAGACAGATTTTTAGTAGAGATGGGGTTTCAC[illegible]	1st PCR amplification
OT11_INTS1_V2_F	TCGTCGGCAGCGTCAGATGTGTATAAGAGACAGGGTTTTACCCTCCTTGACCCTG	1st PCR amplification
OT11_INTS1_V2_R	GTCTCGTGGGCTCGGAGATGTGTATAAGAGACAGCTCTCAGTGATGGTCTGCTTGGT	1st PCR amplification
OT12_GBA3_V2_F	TCGTCGGCAGCGTCAGATGTGTATAAGAGACAGGAGGAAACCTGGATGATGAGATTCC	1st PCR amplification
OT12_GBA3_V2_R	GTCTCGTGGGCTCGGAGATGTGTATAAGAGACAGAAGCCACCTACTCTTTCGTACTTTG	1st PCR amplification
OT13 _E1983471_F	TCGTCGGCAGCGTCAGATGTGTATAAGAGACAGCATCAACAACTGACCCCAAAAGC	1st PCR amplification
OT13 _E1983471_R	GTCTCGTGGGCTCGGAGATGTGTATAAGAGACAGGCTGGGAAGGAATCCAGAGACC	1st PCR amplification
OT14_MX2_V2_F	TCGTCGGCAGCGTCAGATGTGTATAAGAGACAGCCTTTCCTGGGTTCAGTAGCATC	1st PCR amplification
OT14_MX2_V2_R	GTCTCGTGGGCTCGGAGATGTGTATAAGAGACAGTGGAGTCAGCATGTCAGGTGTCT	1st PCR amplification
N701	CAAGCAGAAGACGGCATACGAGATTCGCCTTAGTCTCGTGGGCTCGG	2nd PCR amplification
N702	CAAGCAGAAGACGGCATACGAGATCTAGTACGGTCTCGTGGGCTCGG	2nd PCR amplification
N703	CAAGCAGAAGACGGCATACGAGATTTCTGCCTGTCTCGTGGGCTCGG	2nd PCR amplification
N704	CAAGCAGAAGACGGCATACGAGATGCTCAGGAGTCTCGTGGGCTCGG	2nd PCR amplification
N705	CAAGCAGAAGACGGCATACGAGATAGGAGTCCGTCTCGTGGGCTCGG	2nd PCR amplification
N706	CAAGCAGAAGACGGCATACGAGATCATGCCTAGTCTCGTGGGCTCGG	2nd PCR amplification
N707	CAAGCAGAAGACGGCATACGAGATGTAGAGAGGTCTCGTGGGCTCGG	2nd PCR amplification
N710	CAAGCAGAAGACGGCATACGAGATCAGCCTCGGTCTCGTGGGCTCGG	2nd PCR amplification
N711	CAAGCAGAAGACGGCATACGAGATTGCCTCTTGTCTCGTGGGCTCGG	2nd PCR amplification
N712	CAAGCAGAAGACGGCATACGAGATTCCTCTACGTCTCGTGGGCTCGG	2nd PCR amplification
N714	CAAGCAGAAGACGGCATACGAGATTCATGAGCGTCTCGTGGGCTCGG	2nd PCR amplification
N715	CAAGCAGAAGACGGCATACGAGATCCTGAGATGTCTCGTGGGCTCGG	2nd PCR amplification
S506	AATGATACGGCGACCACCGAGATCTACACACTGCATATCGTCGGCAGCGTC	2nd PCR amplification
S507	AATGATACGGCGACCACCGAGATCTACACAAGGAGTATCGTCGGCAGCGTC	2nd PCR amplification
